# Application of Periodontal Ligament-Derived Multipotent Mesenchymal Stromal Cell Sheets for Periodontal Regeneration

**DOI:** 10.3390/ijms20112796

**Published:** 2019-06-07

**Authors:** Satoru Onizuka, Takanori Iwata

**Affiliations:** 1 Division of Periodontology, Department of Oral Functions, Kyushu Dental University, Fukuoka 803-8580, Japan; r18onizuka@fa.kyu-dent.ac.jp; 2 Institute of Advanced Biomedical Engineering and Science, Tokyo Women’s Medical University, Tokyo 162-8666, Japan; 3 Department of Periodontology, Graduate School of Medical and Dental Sciences, Tokyo Medical and Dental University, Tokyo 113-8549, Japan

**Keywords:** periodontal ligament, stem cells, MSCs, periodontal regeneration, clinical study

## Abstract

Periodontitis is a chronic inflammatory disorder that causes destruction of the periodontal attachment apparatus including alveolar bone, the periodontal ligament, and cementum. Dental implants have been routinely installed after extraction of periodontitis-affected teeth; however, recent studies have indicated that many dental implants are affected by peri-implantitis, which progresses rapidly because of the failure of the immune system. Therefore, there is a renewed focus on periodontal regeneration aroundnatural teeth. To regenerate periodontal tissue, many researchers and clinicians have attempted to perform periodontal regenerative therapy using materials such as bioresorbable scaffolds, growth factors, and cells. The concept of guided tissue regeneration, by which endogenous periodontal ligament- and alveolar bone-derived cells are preferentially proliferated by barrier membranes, has proved effective, and various kinds of membranes are now commercially available. Clinical studies have shown the significance of barrier membranes for periodontal regeneration; however, the technique is indicated only for relatively small infrabony defects. Cytokine therapies have also been introduced to promote periodontal regeneration, but the indications are also for small size defects. To overcome this limitation, ex vivo expanded multipotent mesenchymal stromal cells (MSCs) have been studied. In particular, periodontal ligament-derived multipotent mesenchymal stromal cells are thought to be a responsible cell source, based on both translational and clinical studies. In this review, responsible cell sources for periodontal regeneration and their clinical applications are summarized. In addition, recent transplantation strategies and perspectives about the cytotherapeutic use of stem cells for periodontal regeneration are discussed.

## 1. Introduction

**Figure 1:**
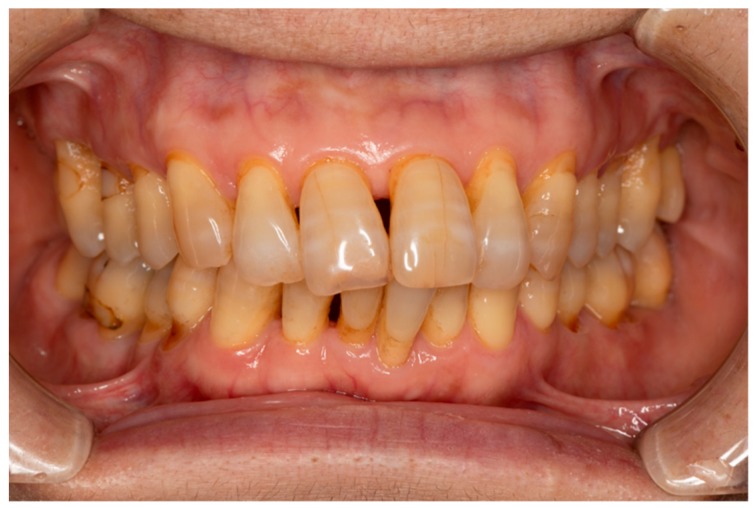
Typical clinical appearance after conventional periodontal treatment. Black triangles appear because of the gingival recessions, resulting in both functional and aesthetic problems. Gingival recessions occurred in all dentitions, resulting in hypersensitivity and root caries.

Periodontal disease is mainly caused by oral bacteria. Without dental treatment, bacteria-induced inflammation can spread and destroy the periodontal ligament, alveolar bone, cementum, and gingiva. When the destruction of alveolar bone is evident radiographically, it is diagnosed as periodontitis, which is generally considered an irreversible condition. Once periodontitis occurs, it does not heal spontaneously. Therefore, gingival recession usually occurs followed by functional and esthetic problems, such as root caries and black triangles (Figure 1). Moreover, periodontitis not only leads to esthetic and functional problems, but is also associated with systemic diseases such as diabetes, cardiovascular disease, stroke, preterm birth, and pulmonary disease [1]. Thus, periodontitis is an important public health issue, and the development of efficacious therapies to treat periodontitis should be a major goal of the health sciences. To overcome these problems, periodontal regeneration has been studied for almost 100 years. To our knowledge, the first report of periodontal regeneration [2] was published in 1923 in relation to autologous bone transplantation. Since then, various kind of bone substrates, not only autologous but also allogenic, xenogeneic, and synthesized materials have been studied for use in periodontal regeneration, and their efficacy has been systematically reviewed [3]. Generally speaking, autologous bone is a superior substrate to others, and the bigger the defect size is the less effective these bone substrates will work. Many of these materials are commercially available and clinically effective, although histological results have not shown true periodontal regeneration, which would include newly formed cementum and well-oriented periodontal fibers. To induce true periodontal regeneration, the concept of guided tissue regeneration (GTR) was introduced in the 1980s, with the use of occlusive membranes to eliminate the downgrowth of epithelial cells, resulting in preferential proliferation of cells favorable for periodontal regeneration such as periodontal ligament cells and osteoblasts [4]. This strategy was momentous because cell migration was controlled by a barrier membrane based on the biological wound healing process. Biologically active regenerative materials have been studied since the 1990s, and some products, such as enamel matrix derivative, platelet-derived growth factor (PDGF)-BB, and fibroblast growth factor (FGF)-2, have been approved for clinical use. These biologically active regenerative materials are thought to function by controlling the wound healing process at surgical sites via cellular migration, proliferation, and differentiation.

Because the regenerative therapies mentioned above have limited indications and do not provide good results for a wide range of defects, such as one-wall infrabony defects, class III furcation defects, and horizontal defects, cytotherapeutic approaches were introduced in the 2000s. Based on recent developments in stem cell biology and tissue engineering, stem cells from patients or healthy volunteer donors can be harvested and amplified in vitro. Stem cells can then be manufactured with or without scaffolds and transplanted for periodontal regeneration.

## 2. Current Cytotherapy for Periodontal Regeneration in Humans

It is thought that there are two main modes of action of cytotherapy in periodontal regeneration. One is the supply of favorable cells for periodontal regeneration, such as periodontal ligament cells and/or osteoblastic cells. The other is the support of endogenous favorable cells through the paracrine effects of transplanted cells. Sometimes these two modes of actions are combined.

This section reviews the current literature about cytotherapy, mainly using multipotent mesenchymal stromal cells, for periodontal regeneration.

### 2.1. Multipotent Mesenchymal Stromal Cells (MSCs)

Multipotent mesenchymal stromal cells (MSCs) can be isolated from several tissues including bone marrow, fat, periodontal ligament, dental pulp, and periosteum. Because the MSCs used in each laboratory were different, the MSC Committee of the International Society of Cell Therapy (ISCT) published a statement paper in 2006 that defined the minimal criteria of human MSC characteristics, including the following: (1) adherence to plastic under standard culture conditions; (2) specific surface antigen expression (positive for CD105, CD73, and CD90; negative for CD45, CD34, CD14 or CD11b, CD79a or CD19, and HLA-DR); and (3) in vitro differentiation capacity into osteoblasts, adipocytes, and chondroblasts [5]. Although these cells exhibit different characteristics in terms of proliferation ability, differentiation potential, and gene expression profiles, most of the cells possess additional characteristics, such as immunomodulatory properties, and the ability to mediate trophic factors [6]. Because of these secretions from MSCs, transplantation of MSCs has been clinically performed all over the world for immune- and inflammation-mediated diseases in which steroid treatment does not work well [7]. In this section, we discuss MSCs derived from several types of tissue used for periodontal regenerative therapy.

#### 2.1.1. Periodontal Ligament (PDL)-Derived Cells

Periodontal ligament (PDL) tissue is the soft connective tissue between the tooth and the alveolar bone. PDL tissue mainly consists of fibroblastic ligament cells that function as a cushion at the time of chewing. PDL tissue also contains abundant nerves and vessels and, thus, transmits information about food texture to the brain and retains an immune response against oral bacteria. Clinically, PDL tissue is important in autotransplantation because the PDL tissue of the donor’s teeth has the potential to induce the formation of new bone, gingiva, and PDL at the recipient site [8]. In fact, postnatal stem cells were discovered in PDL tissue and termed PDL stem cells (PDLSCs) by Dr. Shi and colleagues [9], and a transplantation study showed that human PDLSCs could differentiate into periodontal ligament, cementum, and alveolar bone cells in immunocompromised beige mice. In this report, PDLSCs were defined as being double positive for STRO-1/CD146, but there was no mention of whether PDLSCs possessed chondrogenic potential. In contrast, many researchers have focused on the MSC-like population in PDL tissue and determined that PDL tissue-derived cells had the characteristics of MSCs [10,11,12,13]. Trubiani et al. [14] named these cells PDL-MSCs. Our research group also targeted the multipotency of PDL-MSCs. Many samples showed the capacity for differentiation into both osteoblasts and adipocytes, with little chondrogenic potential [13]. We hypothesize that PDL-MSCs have high alkaline phosphatase activity without osteoinductive treatment; therefore, they possess high potential for differentiation into osteoblasts. Surprisingly, PDL-MSCs express both osteoblastic and known periodontal marker genes, such as periostin and S100A4, when they are cultured in an osteoinductive medium, suggesting that osteoinductive pretreatment may enhance the ability of PDL-MSCs to induce periodontal regeneration.

#### 2.1.2. Cell Sheet Engineering Technology for PDL-MSCs

It is important that ex vivo expanded MSCs are transplanted in an appropriate position. The injection of a single cell suspension is known to be inefficient because it is unstable and will diffuse. Our research group introduced cell sheet engineering technology, using temperature-responsive cell culture dishes, in which cells can be harvested in a sheet form [15]. We developed a temperature-responsive polymer named *N*-isopropylacrylamide (PIPAAm) for the surface of cell culture dishes. At temperatures lower than 32 °C, PIPAAm is fully hydrated with an extended chain conformation; however, at temperatures higher than 32 °C, PIPAAm is extensively dehydrated and compact. Cells generally adhere to hydrophobic surfaces but not to highly hydrated hydrophilic surfaces. Our laboratory grafted PIPAAm to develop temperature-responsive culture dishes by irradiation with an electron beam. The advantage of a temperature-responsive dish is the possibility of harvesting intact cells and proteins with only low-temperature treatment. By reducing the temperature, cells spontaneously detach from the surface of dishes. Compared with the process of retrieving cells with enzymes such as trypsin or dispase, loss of cell viability and degradation of surface proteins are minimized. Recent reports have clearly demonstrated that various kinds of single cells harvested from temperature-responsive dishes have highly preserved functions as well as intact proteins. Additionally, the preserved subcellular matrix proteins of harvested cell sheets provide adhesive properties for stacking. Therefore, cell sheets can be stabilized at the recipient sites without any glue or sutures in a corneal reconstruction model [16]. It is also possible to create thick tissues by layering multiple cell sheets.

PDL cell sheets were manufactured and transplanted, then periodontal regeneration was observed in two animal models [17,18]. Following translational studies, including large animal studies [19,20,21,22], characterization of human PDL-derived multipotent mesenchymal stromal cells (hPDL-MSCs) [13,23,24,25], and safety and efficacy tests of hPDL-MSCs [15,26,27,28], a clinical study of periodontal regeneration with autologous periodontal ligament cell sheets started in 2011. Autologous PDL-MSCs were isolated from 10 patients’ own redundant teeth. Cells were expanded for two weeks and cultured with osteoinductive supplements for an additional two weeks on temperature-responsive culture dishes in the cell processing center. Then, three-layered PDL-MSC sheets were created and transplanted on the cleaned root surface. Bony defects were filled with beta-tricalcium phosphate granules.

The results of this clinical study validated the safety and efficacy of autologous PDL-MSC sheets in severe periodontal defects. All the findings, including reduced periodontal probing depth (mean ± SD, 3.2 ± 1.9 mm), clinical attachment gain (2.5 ± 2.6 mm), and increased radiographic bone height (2.3 ± 1.8 mm), indicated improved periodontal conditions in all 10 cases at 6 months after the transplantation. These therapeutic effects were sustained during a mean follow-up period of 55 ± 19 months, and there were no serious adverse events [29].

#### 2.1.3. Transplantation of PDL Stem Cell (PDLSC) Sheet-Wrapped Bone Substrates

A randomized clinical trial of autologous PDLSCs with GTR membranes and bovine-derived bone substrates was reported in 2016 [30] based on a previous case report study [31]. The obtained PDL tissue was dissociated in a solution of 0.2% collagenase type I, and PDLSCs were cultured for expansion. The PDLSCs were digested by trypsin to obtain single cell suspensions and were inoculated on 6-well plates at 1 × 10^5^ cells/well with 30 μg/mL of l-ascorbic acid for approximately 10 days. PDLSC sheets were mechanically detached, rolled up to pack the xenogeneic bone substrates (Bio-Oss^®^), and placed 1 mm over the crest height of the defects. Collagen membrane (Bio-Guide^®^) was placed on the PDLSC sheet/bone substrate complex to cover. The authors observed the safety of the autologous PDLSCs, although they did not find any significant therapeutic efficacy of the PDLSCs compared with that of the control group (collagen membrane and bone substrate without cells). It was possible that the bone substrates used in this clinical trial may have masked the potential of the PDLSC sheet because they were considered to be slow-resorbing materials. Further research should be undertaken with mechanically detached PDLSC sheets to clarify this point.

As mentioned above, PDL-derived cells may have the potential to induce true periodontal regeneration around the natural teeth. However, there is no standard protocol for isolation and cultivation of PDL-derived cells. It may be important to define the optimal culture conditions and an evaluating system for PDL-derived cells. Additional research should be undertaken to determine the characteristics of transplanted cells for further development of cytotherapy with PDL-derived cells.

### 2.2. Other Tissue-Derived Cells

Iliac bone marrow MSCs (BMMSCs) are a well-characterized cell source for bone regeneration. BMMSCs combined with biomaterials have been studied in relation to periodontal regeneration. BMMSCs mixed with platelet-rich plasma (PRP) were transplanted in 17 patients, and reduced probing depth, clinical attachment gain, and bone gain were observed, although the defect shape was not mentioned [32].

Periosteal cells possess the capacity for bone regeneration, and they are easily harvested. Membranous cultured periosteum was transplanted in the same way as the GTR membrane, and its safety and efficacy were confirmed [33,34].

Recently, a clinical study with a single-cell suspension of adipose stem cells with fibrin glue was undertaken in Osaka, Japan. Abdominal fat tissue was harvested from each patient and cultured under specific culture conditions. The results have not yet been disclosed.

Mechanically dissociated dental pulp tissue has been used in clinical settings [35,36]. Although the characteristics of micrografts were not mentioned in detail, a randomized controlled clinical trial showed significant efficacy of autologous uncultured dental pulp micrografts on periodontal parameters one year after surgery [37]. Table 1 summarizes the ex vivo expanded stem/progenitor cell transplantation studies for periodontal regeneration in clinical settings. Various types of cells combined with different biomaterials were transplanted into a range of defects. Cytotherapeutic clinical research has only recently started; however, consensus must be established in terms of cell characterization, bone substrates, defect shapes, and evaluation systems with the cooperation of researchers all over the world.

## 3. Recent Progress of Tissue Engineering for Periodontal Regeneration

This section reviews the recent progress of tissue engineering for periodontal regeneration.

### 3.1. Intelligent Scaffolds

Recent polymer engineering has enabled the development of scaffolds with functions. Cell orientation and cell type can be controlled by cell culture surface modification [38] or polymer design [39]. A three-dimensional printed bioresorbable scaffold for periodontal regeneration was designed and transplanted with rhPDGF-BB [40]. The authors reported that the treated site remained intact for 12 months following therapy.

### 3.2. Scaffold-Free Culture Technique

In addition to the cell sheet engineering technology mentioned above, various cell manufacturing technologies have been established. A spheroid culture technique was introduced for PDL-MSCs with enhanced osteogenic potential [41]; therefore, this technique may be effective for hard tissue regeneration. Another study suggested that spheroid culture affected several characteristics of PDLSCs, including the expression of genes related to anti-inflammation and angiogenesis. It is thought that apoptosis signaling may be involved in these changes [42]. These studies suggest that culture conditions alter the characteristics of PDL-MSCs.

### 3.3. Decellularized Tissue-Engineered Constructs

Recently, decellularized materials have been introduced for periodontal regeneration. hPDL cell sheets were chemically decellularized by NH_4_OH/Triton X-100 and DNase solutions, resulting in an ideal scaffold, with intact extracellular matrices and resident growth factors, and that can support repopulation by allogeneic cells [43]. The same research group also decellularized the PDL cell sheet construct, which was loaded on polycaprolactone scaffolds and then transplanted into rat periodontal defect models [44]. The decellularized construct without vital cells enhanced the formation of attachment, suggesting that this construct itself enhanced periodontal regeneration. This product may, therefore, have the potential to be an off-the-shelf product.

## 4. Perspective of Future Cytotherapy for Periodontal Regeneration

### 4.1. Allogeneic Cell Sources and Establishment of Cell Banks

Our autologous transplantation study of PDL-MSC sheets proved their safety and feasibility for clinical use [29]. However, some of the problems of this strategy have become clear. First, patients who do not have redundant teeth cannot receive this treatment because of the lack of a cell source. Almost all older patients do not have such teeth because their wisdom teeth may have already been extracted in their youth. Second, even in patients with redundant teeth, there is a relatively large individual variation in cell proliferation and phenotype. Therefore, it is hard to produce consistent and stable products. Third, it takes about four weeks to produce this cellular product from the tooth, making it an expensive process. Additionally, considering the large number of patients with periodontitis, it is difficult to produce many autologous products at the same time because of the limited capacity of the cell processing center. Therefore, we shifted our strategy from autologous to allogeneic PDL-MSC transplantation to treat a greater number of patients at a lower cost.

Because wisdom teeth are routinely extracted from young patients, there is a limitless source of cells to establish a cell bank of allogeneic PDL-MSCs. Extracted teeth were collected, and the periodontal ligament tissue of each tooth was dissected out. MSCs were isolated, cultured, and stocked at passage three as master cell banks. The master cell banks were thawed and cultured to construct working cell banks at passage five. Working cell banks were further cultured to make PDL-MSC sheets on temperature-responsive culture dishes. PDL-MSCs were seeded on temperature-responsive culture dishes at a cell density of (3 × 10^5^)–(4 × 10^5^) cells/dish and cultured in an osteoinductive medium (complete medium with additional 82.1 μg/mL l-ascorbic acid phosphate magnesium salt *N*-hydrate, 10 nM dexamethasone, and 10 mM β-glycerophosphate) for 9–10 day. Culturing PDL-MSCs with osteoinductive supplements enhances bone regeneration in vivo [19] and expression of both osteoblastic and periodontal marker genes in vitro [19,45], suggesting that the osteoinductive medium may improve the ability of PDL-MSCs to induce periodontal regeneration. We have since established seven different cell banks of PDL-MSCs from young donors aged under 24 years. It is known that younger cells have a higher capacity for proliferation and differentiation [46]. Furthermore, MSCs have been considered hypoimmunogenic because of their limited MHC I expression, lack of MHC II expression, and their ability to modulate immunological responses via T-cell suppression. Therefore, allogeneic MSCs are suitable for use in clinical applications. However, a major concern in using an allogeneic cell source is the risk of infection by donor-derived bacteria and viruses. To confirm the absence of bacterial and viral infection, we screened the donors through interviews and blood tests. Additional blood tests were performed six months after the first examination with the tooth extraction in order to take the window period into account. We then performed various tests for the master cell bank according to rules such as the ICH-Q5A/D. The final tissue and cellular products were then subjected to bacterial, mycoplasma, and virus tests. Therefore, a future task for researchers is to develop a method for evaluating the safety and efficacy of these products at a low cost.

### 4.2. Embryonic Stem/Induced Pluripotent (ES/iPS) Cells

Another solution for the establishment of a cell bank is the use of embryonic stem/induced pluripotent (ES/iPS) cells because they are considered to proliferate permanently. Human ES cells derived from the inner cell mass of blastocysts are pluripotent stem cells capable of differentiating into multiple cell lineages. iPS cells possess characteristics similar to those of ES cells and can be created from somatic cells by overexpression of four key transcription factors: Sox2, Klf4, Oct4, and c-Myc. Although the pathway and protocol for inducing PDL cells are not well understood, a method for inducing MSCs via neural crest cells from ES/iPS cells has been established [47]. The developmental origin of PDL cells is the dental follicle cells [48]. Thus, it is important to understand the differentiation pathway from dental follicles to PDL tissues and cells. Recent studies have shown that the stiffness of cultureware can control the differentiation of MSCs [49]. The extracellular environment must also correctly mimic the original conditions.

### 4.3. Conditioned Medium of MSCs

MSCs produce and secrete proteins and extracellular vesicles into the culture medium, and a recent study suggested that the conditioned medium may have regenerative potential. Nagata et al. collected and concentrated the conditioned medium of PDL-MSCs and used it in rat periodontal defect models [50]. Proteomic analysis revealed that extracellular matrix proteins, enzymes, angiogenic factors, growth factors, and cytokines were contained in the conditioned medium of PDL-MSCs. Furthermore, the administration of conditioned medium of PDL-MSCs resulted in decreased mRNA levels of tumor necrosis factor-α (TNF-α) in healing periodontal tissues. These results suggested that the conditioned medium of PDL-MSCs enhanced periodontal regeneration by suppressing the inflammatory response through TNF-α production. The mechanisms by which this occurs are not well understood; however, recent studies have suggested that conditioned medium contains not only proteins but also extracellular vesicles (EVs) containing proteins, mRNAs, miRNAs, and DNA. Our group focused on EV fractions from the conditioned medium from human oral mucosal epithelial cells and purified EVs [51]. Administration of the EV fraction enhanced wound healing. Further studies are needed to determine which components within the EV fraction have therapeutic effects for wound healing.

## 5. Conclusions

**Figure 2:**
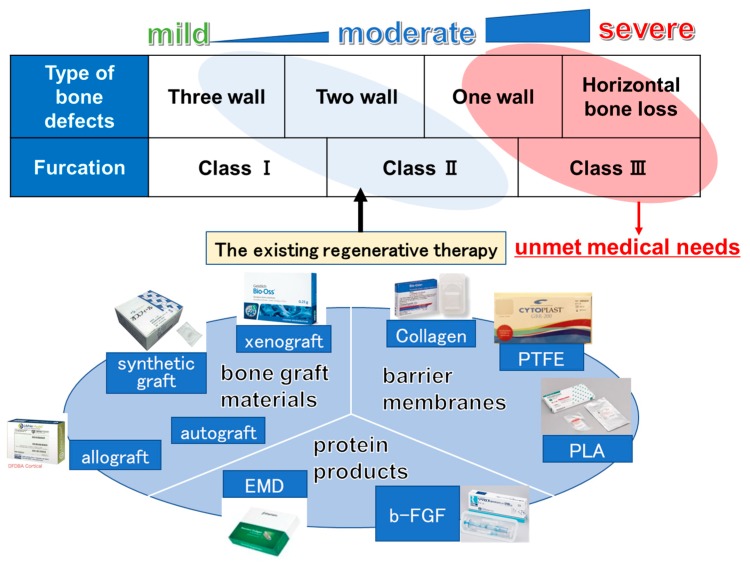
Classification of periodontal bony defects and materials for periodontal regeneration. Conventional regenerative therapies including bone graft materials, protein products, and barrier membrane can be applied to only small size defects such as three-wall defects, two-wall defects, and class II furcation defects.

To date, four clinical studies in which ex vivo expanded stem/progenitor cells were transplanted with biomaterials have been published, and all studies reported the safety and efficacy of their products for periodontal regeneration. Taking into account the concept of GTR, it is clear that the stem cells within the PDL tissue are the key cells for periodontal regeneration and homeostasis; therefore, the cytotherapeutic approach for periodontal regeneration has started with PDL-derived cells. Although many researchers have used MSCs derived from a variety of tissues when using a cytotherapeutic approach for periodontal regeneration, it may be more effective to use MSCs derived from periodontal tissue for cell therapy, considering that they may inherit the characteristics of the tissue of origin. Indeed, PDL-MSCs have shown the greatest potential to regenerate periodontal ligament tissue when compared with other sources of MSCs [20]. In other studies of regenerative therapy, adipose-derived MSCs possessed a high potential for adipogenesis [52], and cartilage-derived MSCs had a high potential for chondrogenesis in an in vitro assay [53]. In light of these considerations, PDL tissue may be the most suitable source of MSCs for periodontal regenerative therapies. However, recent studies also suggest that the transplantation of other kinds of MSCs supports the proliferation and differentiation of endogenous PDL cells via a paracrine effect. Therefore, these two modes of action are proposed for periodontal regeneration. Additionally, recent animal studies have shown that decellularized extracellular matrix (ECM) products without cells can enhance periodontal regeneration, suggesting that ECMs secreted by cells can act as an additional mode of action for periodontal regeneration. Further investigation into this area is required. In terms of allogeneic transplantation, a recent review mentioned that MSCs have an immune-modulatory effect [6]; therefore, few strong signs of immunological rejection have been observed. In fact, when we transplanted allogeneic PDL-MSCs in a canine model, the transplanted cells had almost disappeared at eight weeks after the surgery with no adverse events [54]. We started a phase I/IIa safety and efficacy study of an allogeneic periodontal ligament-derived mesenchymal stromal cell sheet product (TWP-0001) in patients with periodontitis, consisting of one-wall infrabony defects, class III furcation defects, or horizontal defects, with probing depths of 4–9 mm after the initial therapy. Conventional regenerative therapies have already overcome small size defects, such as three-wall defects, two-wall defects, and class II furcation defects, so cell products are not required for these cases. The cost of cytotherapy is relatively high; therefore, we are targeting patients with severe periodontal defects that have long been untreatable (Figure 2). However, to achieve periodontal regeneration, it is important to firstly diagnose whether the condition is periodontitis or a related condition. Several studies have suggested that new technologies, such as cone-beam computed tomography (CBCT) and DNA methylation of specific genes, can be used as diagnostic tools for oral diseases [55,56,57]. Hence, further research is required not only into periodontal regeneration, but also into diagnostic tools to assess the periodontal status. If our cytotherapy technique proves to be effective, it will improve the retention rate for natural teeth and will reduce the need for dental implants.

The field of regenerative medicine is rapidly evolving through the fusion of stem cell biology and tissue engineering. Transplantation of MSCs is under trial not only for regenerative medicine but also in clinical applications for intractable diseases such as graft versus host rejection (GVHD) in expectation of its immune-regulatory function, and it has been approved in some countries. It is clear that an allogeneic approach is essential for the widespread use of regenerative therapy. We also investigated the allogeneic use of PDL-MSCs, and a clinical trial began in 2018. It is important to consider not only the efficacy, but also the safety, of cellular products, and industry-academia collaboration is necessary to expand this treatment. Although verification by clinical trials has not yet occurred, it is hoped that hMSC can assist in addressing unmet medical needs.

## Figures and Tables

**Table 1:** Ex vivo expanded stem/progenitor cell transplantation studies for periodontal regeneration in clinical settings.

Reference #	Cells	Other Materials	Stem Cells	Patient #	Defect Shape	Results (mm) Gain of LBH
Iwata	Periodontal Ligament Mesenchymal Stromal Cells (PDL-MSCs)	woven PGA mesh beta-TCP	yes	10	8 severe defects 2 two or three-wall defects	2.3 ± 1.8 (6M)
Chen	Periodontal Ligament Stem Cells (PDLSCs)	Guided Tissue Regeneration (GTR) membrane bovine-derived bone substrates	yes	20	-	2.31* (3M) 2.59 *(6M) 2.71*(12M)
Yamada	Iliac Bone Marrow Mesenchymal Stromal Cells (BMMSCs)	Platelet-Rich Plasma (PRP)	yes	17	-	3.12 ± 1.23 (12M)
Yamamiya	Periosteum-derived cells	PRP hydroxyapatite	unknown	15	2 severe defects13 two or three-wall defects	4.9 ± 1.2 (12M)

Severe defects consist of one-wall intrabony defects, Class III furcation defects, and horizontal defects. *: Authors of this review calculated from the original paper. LBH; liner bone height.

## References

[1] Pihlstrom B.L., Michalowicz B.S., Johnson N.W. (2005). Periodontal diseases. Lancet.

[2] Hegedus Z. (1923). The rebuilding of the alveolar processes by bone transplantation. Dent. Cosmos..

[3] Sculean A., Nikolidakis D., Nikou G., Ivanovic A., Chapple I.L., Stavropoulos A. (2015). Biomaterials for promoting periodontal regeneration in human intrabony defects: A systematic review. Periodontol 2000.

[4] Bartold P.M., Gronthos S., Ivanovski S., Fisher A., Hutmacher D.W. (2016). Tissue engineered periodontal products. J. Periodontal Res..

[5] Dominici M., Le Blanc K., Mueller I., Slaper-Cortenbach I., Marini F., Krause D., Deans R., Keating A., Prockop D., Horwitz E. (2006). Minimal criteria for defining multipotent mesenchymal stromal cells. The International Society for Cellular Therapy position statement. Cytotherapy.

[6] Caplan A.I. (2017). Mesenchymal Stem Cells: Time to Change the Name!. Stem Cells Transl. Med..

[7] Wang L.T., Ting C.H., Yen M.L., Liu K.J., Sytwu H.K., Wu K.K., Yen B.L. (2016). Human mesenchymal stem cells (MSCs) for treatment towards immune- and inflammation-mediated diseases: Review of current clinical trials. J. Biomed. Sci..

[8] Tsukiboshi M. (2002). Autotransplantation of teeth: Requirements for predictable success. Dent. Traumatol..

[9] Seo B.M., Miura M., Gronthos S., Bartold P.M., Batouli S., Brahim J., Young M., Robey P.G., Wang C.Y., Shi S. (2004). Investigation of multipotent postnatal stem cells from human periodontal ligament. Lancet.

[10] Gay I.C., Chen S., MacDougall M. (2007). Isolation and characterization of multipotent human periodontal ligament stem cells. Orthod. Craniofac. Res..

[11] Lindroos B., Maenpaa K., Ylikomi T., Oja H., Suuronen R., Miettinen S. (2008). Characterisation of human dental stem cells and buccal mucosa fibroblasts. Biochem. Biophys. Res. Commun..

[12] Xu J., Wang W., Kapila Y., Lotz J., Kapila S. (2009). Multiple differentiation capacity of STRO-1+/CD146+ PDL mesenchymal progenitor cells. Stem Cells Dev..

[13] Iwata T., Yamato M., Zhang Z., Mukobata S., Washio K., Ando T., Feijen J., Okano T., Ishikawa I. (2010). Validation of human periodontal ligament-derived cells as a reliable source for cytotherapeutic use. J. Clin. Periodontol..

[14] Trubiani O., Di Primio R., Traini T., Pizzicannella J., Scarano A., Piattelli A., Caputi S. (2005). Morphological and cytofluorimetric analysis of adult mesenchymal stem cells expanded ex vivo from periodontal ligament. Int. J. Immunopathol. Pharmacol..

[15] Iwata T., Washio K., Yoshida T., Ishikawa I., Ando T., Yamato M., Okano T. (2015). Cell sheet engineering and its application for periodontal regeneration. J. Tissue Eng. Regen. Med..

[16] Nishida K., Yamato M., Hayashida Y., Watanabe K., Yamamoto K., Adachi E., Nagai S., Kikuchi A., Maeda N., Watanabe H. (2004). Corneal reconstruction with tissue-engineered cell sheets composed of autologous oral mucosal epithelium. N. Engl. J. Med..

[17] Hagiwara S., Okano T., Nakano S., Nagaoka S., Hasegawa K., Yamaguchi S. (1996). Preventive treatment of gastroesophageal varices. Nippon Geka Gakkai Zasshi.

[18] Akizuki T., Oda S., Komaki M., Tsuchioka H., Kawakatsu N., Kikuchi A., Yamato M., Okano T., Ishikawa I. (2005). Application of periodontal ligament cell sheet for periodontal regeneration: A pilot study in beagle dogs. J. Periodontal. Res..

[19] Iwata T., Yamato M., Tsuchioka H., Takagi R., Mukobata S., Washio K., Okano T., Ishikawa I. (2009). Periodontal regeneration with multi-layered periodontal ligament-derived cell sheets in a canine model. Biomaterials.

[20] Tsumanuma Y., Iwata T., Washio K., Yoshida T., Yamada A., Takagi R., Ohno T., Lin K., Yamato M., Ishikawa I. (2011). Comparison of different tissue-derived stem cell sheets for periodontal regeneration in a canine 1-wall defect model. Biomaterials.

[21] Izumi Y., Aoki A., Yamada Y., Kobayashi H., Iwata T., Akizuki T., Suda T., Nakamura S., Wara-Aswapati N., Ueda M. (2011). Current and future periodontal tissue engineering. Periodontol. 2000.

[22] Iwata T., Yamato M., Ishikawa I., Ando T., Okano T. (2014). Tissue engineering in periodontal tissue. Anatomical. Record..

[23] Ishikawa I., Iwata T., Washio K., Okano T., Nagasawa T., Iwasaki K., Ando T. (2009). Cell sheet engineering and other novel cell-based approaches to periodontal regeneration. Periodontol. 2000.

[24] Yamada A., Iwata T., Yamato M., Okano T., Izumi Y. (2013). Diverse functions of secreted frizzled-related proteins in the osteoblastogenesis of human multipotent mesenchymal stromal cells. Biomaterials.

[25] Suphanantachat S., Iwata T., Ishihara J., Yamato M., Okano T., Izumi Y. (2014). A role for c-Kit in the maintenance of undifferentiated human mesenchymal stromal cells. Biomaterials.

[26] Washio K., Iwata T., Mizutani M., Ando T., Yamato M., Okano T., Ishikawa I. (2010). Assessment of cell sheets derived from human periodontal ligament cells: A pre-clinical study. Cell Tissue Res..

[27] Yoshida T., Washio K., Iwata T., Okano T., Ishikawa I. (2012). Current status and future development of cell transplantation therapy for periodontal tissue regeneration. Int. J. Dent..

[28] Washio K., Kuroda H., Iwata T., Yoshida T., Yamato M., Okano T. (2014). Improved enzymatic treatment for accurate cell counting from extracellular matrix-rich periodontal ligament cell sheets. Int. J. Oral Maxillofac. Implants.

[29] Iwata T., Yamato M., Washio K., Yoshida T., Tsumanuma Y., Yamada A., Onizuka S., Izumi Y., Ando T., Okano T. (2018). Periodontal regeneration with autologous periodontal ligament-derived cell sheets—A safety and efficacy study in ten patients. Regen. Ther..

[30] Chen F.M., Gao L.N., Tian B.M., Zhang X.Y., Zhang Y.J., Dong G.Y., Lu H., Chu Q., Xu J., Yu Y. (2016). Treatment of periodontal intrabony defects using autologous periodontal ligament stem cells: A randomized clinical trial. Stem Cell Res. Ther..

[31] Feng F., Akiyama K., Liu Y., Yamaza T., Wang T.M., Chen J.H., Wang B.B., Huang G.T., Wang S., Shi S. (2010). Utility of PDL progenitors for in vivo tissue regeneration: A report of 3 cases. Oral Dis..

[32] Yamada Y., Nakamura S., Ito K., Umemura E., Hara K., Nagasaka T., Abe A., Baba S., Furuichi Y., Izumi Y. (2013). Injectable bone tissue engineering using expanded mesenchymal stem cells. Stem Cells.

[33] Okuda K., Yamamiya K., Kawase T., Mizuno H., Ueda M., Yoshie H. (2009). Treatment of human infrabony periodontal defects by grafting human cultured periosteum sheets combined with platelet-rich plasma and porous hydroxyapatite granules: Case series. J. Int. Acad. Periodontol..

[34] Yamamiya K., Okuda K., Kawase T., Hata K., Wolff L.F., Yoshie H. (2008). Tissue-engineered cultured periosteum used with platelet-rich plasma and hydroxyapatite in treating human osseous defects. J. Periodontol..

[35] Aimetti M., Ferrarotti F., Cricenti L., Mariani G.M., Romano F. (2014). Autologous dental pulp stem cells in periodontal regeneration: A case report. Int. J. Periodontics Restorative Dent..

[36] Aimetti M., Ferrarotti F., Gamba M.N., Giraudi M., Romano F. (2018). Regenerative Treatment of Periodontal Intrabony Defects Using Autologous Dental Pulp Stem Cells: A 1-Year Follow-Up Case Series. Int. J. Periodontics Restorative Dent..

[37] Ferrarotti F., Romano F., Gamba M.N., Quirico A., Giraudi M., Audagna M., Aimetti M. (2018). Human intrabony defect regeneration with micrografts containing dental pulp stem cells: A randomized controlled clinical trial. J. Clin. Periodontol..

[38] Takahashi H., Nakayama M., Shimizu T., Yamato M., Okano T. (2011). Anisotropic cell sheets for constructing three-dimensional tissue with well-organized cell orientation. Biomaterials.

[39] Park C.H., Rios H.F., Jin Q., Bland M.E., Flanagan C.L., Hollister S.J., Giannobile W.V. (2010). Biomimetic hybrid scaffolds for engineering human tooth-ligament interfaces. Biomaterials.

[40] Rasperini G., Pilipchuk S.P., Flanagan C.L., Park C.H., Pagni G., Hollister S.J., Giannobile W.V. (2015). 3D-printed Bioresorbable Scaffold for Periodontal Repair. J. Dent. Res..

[41] Moritani Y., Usui M., Sano K., Nakazawa K., Hanatani T., Nakatomi M., Iwata T., Sato T., Ariyoshi W., Nishihara T. (2018). Spheroid culture enhances osteogenic potential of periodontal ligament mesenchymal stem cells. J. Periodontal. Res..

[42] Iwasaki K., Nagata M., Akazawa K., Watabe T., Morita I. (2018). Changes in characteristics of periodontal ligament stem cells in spheroid culture. J. Periodontal. Res..

[43] Farag A., Vaquette C., Theodoropoulos C., Hamlet S.M., Hutmacher D.W., Ivanovski S. (2014). Decellularized periodontal ligament cell sheets with recellularization potential. J. Dent. Res..

[44] Farag A., Hashimi S.M., Vaquette C., Bartold P.M., Hutmacher D.W., Ivanovski S. (2018). The effect of decellularized tissue engineered constructs on periodontal regeneration. J. Clin. Periodontol..

[45] Onizuka S., Iwata T., Park S.J., Nakai K., Yamato M., Okano T., Izumi Y. (2016). ZBTB16 as a Downstream Target Gene of Osterix Regulates Osteoblastogenesis of Human Multipotent Mesenchymal Stromal Cells. J. Cell Biochem..

[46] Ye X., Liao C., Liu G., Xu Y., Tan J., Song Z. (2016). Age-Related Changes in the Regenerative Potential of Adipose-Derived Stem Cells Isolated from the Prominent Fat Pads in Human Lower Eyelids. PLoS ONE.

[47] Fukuta M., Nakai Y., Kirino K., Nakagawa M., Sekiguchi K., Nagata S., Matsumoto Y., Yamamoto T., Umeda K., Heike T. (2014). Derivation of mesenchymal stromal cells from pluripotent stem cells through a neural crest lineage using small molecule compounds with defined media. PLoS ONE.

[48] Fleischmannova J., Matalova E., Sharpe P.T., Misek I., Radlanski R.J. (2010). Formation of the tooth-bone interface. J. Dent. Res..

[49] Lv H., Li L., Sun M., Zhang Y., Chen L., Rong Y., Li Y. (2015). Mechanism of regulation of stem cell differentiation by matrix stiffness. Stem Cell Res. Ther..

[50] Nagata M., Iwasaki K., Akazawa K., Komaki M., Yokoyama N., Izumi Y., Morita I. (2017). Conditioned Medium from Periodontal Ligament Stem Cells Enhances Periodontal Regeneration. Tissue Eng. Part A.

[51] Sjoqvist S., Ishikawa T., Shimura D., Kasai Y., Imafuku A., Bou-Ghannam S., Iwata T., Kanai N. (2019). Exosomes derived from clinical-grade oral mucosal epithelial cell sheets promote wound healing. J. Extracell. Vesicles.

[52] Lotfy A., Salama M., Zahran F., Jones E., Badawy A., Sobh M. (2014). Characterization of mesenchymal stem cells derived from rat bone marrow and adipose tissue: A comparative study. Int. J. Stem Cells.

[53] Peng L., Jia Z., Yin X., Zhang X., Liu Y., Chen P., Ma K., Zhou C. (2008). Comparative analysis of mesenchymal stem cells from bone marrow, cartilage, and adipose tissue. Stem Cells Dev..

[54] Tsumanuma Y., Iwata T., Kinoshita A., Washio K., Yoshida T., Yamada A., Takagi R., Yamato M., Okano T., Izumi Y. (2016). Allogeneic Transplantation of Periodontal Ligament-Derived Multipotent Mesenchymal Stromal Cell Sheets in Canine Critical-Size Supra-Alveolar Periodontal Defect Model. Biores. Open Access.

[55] Isola G., Cicciù M., Fiorillo L., Matarese G. (2017). Association between Odontoma and Impacted Teeth. J. Craniofac. Surg..

[56] Ferlazzo N., Currò M., Zinellu A., Caccamo D., Isola G., Ventura V., Carru C., Matarese G., Ientile R. (2017). Influence of MTHFR Genetic Background on p16 and MGMT Methylation in Oral Squamous Cell Cancer. Int. J. Mol. Sci..

[57] Raucci G., Pachêco-Pereira C., Grassia V., d’Apuzzo F., Flores-Mir C., Perillo L. (2015). Maxillary arch changes with transpalatal arch treatment followed by full fixed appliances. Angle Orthod..

